# LTβR signalling preferentially accelerates oncogenic AKT-initiated liver tumours

**DOI:** 10.1136/gutjnl-2014-308810

**Published:** 2015-07-23

**Authors:** Anthony J Scarzello, Qun Jiang, Timothy Back, Hien Dang, Deborah Hodge, Charlotte Hanson, Jeffrey Subleski, Jonathan M Weiss, Jimmy K Stauffer, Jitti Chaisaingmongkol, Siritida Rabibhadana, Mathuros Ruchirawat, John Ortaldo, Xin Wei Wang, Paula S Norris, Carl F Ware, Robert H Wiltrout

**Affiliations:** 1 Cancer and Inflamation Program, Center for Cancer Research, National Cancer Institute, Frederick, Maryland, USA; 2 Laboratory of Human Carcinogenesis, Center for Cancer Research, National Cancer Institute, Bethesda, Maryland, USA; 3 Infectious and Inflammatory Diseases Research Center, Sanford Burnham Medical Research Institute, La Jolla, California, USA; 4 Chulabhorn Research Institute, Bangkok, Thailand

**Keywords:** CANCER IMMUNOBIOLOGY

## Abstract

**Objectives:**

The relative contributions of inflammatory signalling and sequential oncogenic dysregulation driving liver cancer pathogenesis remain incompletely understood. Lymphotoxin-β receptor (LTβR) signalling is critically involved in hepatitis and liver tumorigenesis. Therefore, we explored the interdependence of inflammatory lymphotoxin signalling and specific oncogenic pathways in the progression of hepatic cancer.

**Design:**

Pathologically distinct liver tumours were initiated by hydrodynamic transfection of oncogenic V-Akt Murine Thymoma Viral Oncogene Homolog 1 (AKT)/β-catenin or AKT/Notch expressing plasmids. To investigate the relationship of LTβR signalling and specific oncogenic pathways, LTβR antagonist (LTβR-Fc) or agonist (anti-LTβR) were administered post oncogene transfection. Initiated livers/tumours were investigated for changes in oncogene expression, tumour proliferation, progression, latency and pathology. Moreover, specific LTβR-mediated molecular events were investigated in human liver cancer cell lines and through transcriptional analyses of samples from patients with intrahepatic cholangiocarcinoma (ICC).

**Results:**

AKT/β-catenin-transfected livers displayed increased expression of LTβ and LTβR, with antagonism of LTβR signalling reducing tumour progression and enhancing survival. Conversely, enforced LTβR-activation of AKT/β-catenin-initiated tumours induced robust increases in proliferation and progression of hepatic tumour phenotypes in an AKT-dependent manner. LTβR-activation also rapidly accelerated ICC progression initiated by AKT/Notch, but not Notch alone. Moreover, LTβR-accelerated development coincides with increases of Notch, Hes1, c-MYC, pAKT and β-catenin. We further demonstrate LTβR signalling in human liver cancer cell lines to be a regulator of Notch, pAKT_ser473_ and β-catenin. Transcriptome analysis of samples from patients with ICC links increased LTβR network expression with poor patient survival, increased Notch1 expression and Notch and AKT/PI3K signalling.

**Conclusions:**

Our findings link LTβR and oncogenic AKT signalling in the development of ICC.

**Significance of this study**

**What is already known on this subject?**

- Lymphotoxin β receptor (LTβR) inflammatory signalling is upregulated in patients with viral hepatitis and cholangitis and implicated in the initiation of hepatocellular carcinoma (HCC).
- Dysregulation of PI3K/AKT, β-catenin, and Notch pathways are frequently observed in HCC and intrahepatic cholangiocarcinoma (ICC).
- The interconnection between LTβR activation and oncogenic dysregulation towards the development of liver cancer remains incompletely understood.

**What are the new findings?**

- Oncogenic AKT cooperating with β-catenin upregulates LTβ/LTβR to facilitate liver tumour progression.
- LTβR agonism skews AKT/β-catenin pathology towards a more ICC-like phenotype, as well as accelerates oncogenic AKT/Notch-initiated ICC formation in mice. LTβR-mediated tumour progression is dependent on oncogenic AKT and further accelerated when combined with catenin (CAT) or Notch signalling.
- LTβR is widely expressed and maintains oncogene activity in human HCC and ICC cell lines. High levels of LTβR network expression correlates with increased AKT signalling, Notch1 expression as well as poor survival in patients with ICC.

**How might it impact on clinical practice in the foreseeable future?**

- Combination therapies are being extensively explored in preclinical and clinical studies for liver cancer treatment. Combining drugs targeting oncogenic AKT signalling, which have already been in development, with immune agents blocking the activity of the LTβR network may be a valuable new strategy.

## Introduction

Primary liver cancer, consisting of hepatocellular carcinoma (HCC) and intrahepatic cholangiocarcinoma (ICC), is the third leading cause of cancer-related deaths worldwide.^1^ Aetiology-driven liver damage, compensatory proliferation and chronic inflammation culminates in genetic and epigenetic instability, oncogene/tumour suppressor dysregulation^2^ ^3^ and liver cancer formation. AKT, β-catenin and Notch are key oncogenic pathways that are frequently mutated or dysregulated in liver cancer.^3^ However, targeted therapies of these pathways so far have limited efficacy,^4^ in part due to our incomplete understanding of the relative contribution of inflammatory factors and their ability to collaborate with oncogenic pathways. Lymphotoxin β receptor (LTβR) is a member of the tumour necrosis factor (TNF) superfamily of receptors, activated by the proinflammatory cytokines lymphotoxin (LT) αβ heterotrimer and TNFSF14 (LIGHT).^5^ LTαβ expression is primarily restricted to lymphocytes^6^ and is critical for lymph node formation and host defense.^7^ ^8^ LTβR is expressed on most cells with highest expression on epithelial and myeloid lineages.^6^ LTβR signalling is broadly activated during chronic liver inflammation in patients with viral and non-viral hepatitis, cholangitis and HCC.^9^ LTβR signalling in mice has been shown to be critical for liver regeneration,^10^ and lipid homoeostasis.^11^ ^12^ It has also been demonstrated that aberrant expression of LTαβ in hepatocytes is capable of inducing hepatitis and initiating HCC formation in mice through canonical nuclear factor κ-light-chain-enhancer of activated B cells(NF-κB)-dependent mechanisms.^9^ Collectively these studies establish a relationship between LTβR and HCC initiation; however the underlying oncogenic mechanisms driving LTβR-facilitated tumour progression remain incompletely understood. Our efforts are focused on the role of LTβR signalling in modulating oncogenesis using two pathologically distinct models of human liver cancer. Specifically, Sleeping Beauty (SB) mediated transposition,^13^ using plasmids containing oncogenic myristoylated-AKT (AKT)/Δ90β-catenin (CAT) have been shown to initiate liver tumours consisting predominantly of hepatocellular adenoma with some regions of HCC,^14^ while AKT/Notch-intracellular domain (NICD) has been shown to selectively drive ICC.^15^ The novel potential roles of LTβR signalling in ICC were also evaluated in human cholangiocarcinoma cell lines and by comparisons with samples obtained from patients with liver cancer.

## Materials and methods

### Hydrodynamic transfection and Sleeping Beauty plasmids

Liver tumours were initiated in 7–8 week-old female C57/BL6 Jax mice by hydrodynamic tail vein injection of 10% volume/weight of 0.9% saline containing SB third generation (pT3) plasmids expressing oncogenes (transposon) and hyper SB transposase (HBS2) as previously described.^16^ ^13^ AKT/CAT experiments were performed using a transposon:transposase ratio of 10:1 with concentrations of 14 μg/mL mouse myristoylated-AKT, 14 μg/mL human Δ90β-catenin and 3.125 μg/mL HBS2 transposase with constructs obtained as described.^14^ Oncogenic AKT/Notch experiments were performed with 3.125 μg/mL AKT and 12.5 μg/mL mouse NICD and 1.6 μg/mL HBS2. cMET/CAT experiments were performed with 5.625 μg/mL human MET^17^ and 5.625 μg/mL human CAT. Single oncogene experiments were performed using equal amounts of transposon expressing plasmids and pT3 at a 10:1 (transposon:transposase) ratio.

### Gaussia Luciferase assay

Cohydrodynamic transfection of 0.625 μg/mL pT3 containing Gaussia luciferase with oncogene plasmids was used to measure relative oncogene expression and tumour burden as described in *Subleski et al (unpublished, manuscript under review)*. Serum was periodically assayed (1:400) using Dual-Luciferase Reporter Assay (#E1910; Promega) as per manufacturer's instructions.

### Lymphotoxin reagents

Mice were intraperitoneally injected with either 100 μg of agonistic antibody LTβR clone 4H8 (anti-LTβR), 0.1% normal rat serum,100 μg rat IgG (Ig, control),100 μg of soluble decoy receptor, mouse LTβR-Fc (LTβR-Fc) produced in 293T cells and purified by affinity chromatography or 100 μg human IgG (IgG, control) in 200 µL twice per week for durations lasting 4 weeks or 8 weeks. Administration of reagents was initiated on day 10 post hydrodynamic transfection.

### Mouse tissue processing

Mouse livers were either submerged in RNA later (Life Technologies, Gaithersburg, Maryland, USA) for RNA or snap frozen for protein lysate preparations. Livers were also fixed in 10% neutral-buffered formalin phosphate (Fisher Scientific, Pittsburgh, Pennsylvania, USA) to be embedded in paraffin or optimal cutting temperature compound (OCT).

### Immunohistochemistry

The Histology and Tissue Core Facility at the Frederick National Laboratory for Cancer Research routinely performed H&E, Masson's trichrome and immunohistochemical (IHC) staining for LTβR, LTβ, α-feto protein (AFP), CK19, CK8, Notch1, Hes1, pAKT, AKT, Glypican-3, c-MYC, Ki67, β-catenin and CD34 using paraffin sections, and Oil Red O staining with frozen sections. IHC antibodies and methods are listed in online supplementary material. H&E stained liver sections were used to histologically evaluate tumour phenotype/severity. Livers with distinct regions or nodules were counted, with significance determined using Mann-Whitney U test. Livers with less distinct, coalescing lesions were histologically scored with nodule/region distinguished by evidence of compressed margins. Lipogenic hepatic foci were scored as follows: 1 (1–10 nodules) 2 (11–20 nodules) 3 (21–30 nodules) 4 (>30 nodules), hepatoblastoma/HCC-like 1 (1–2 nodules) 2 (3–4 nodules)_3 (5–6 nodules) 4 (>7 nodules), cholangiocyte proliferation /dysplasia 1 (1–3 nodules) 2 (4–7 nodules) 3 (8–11 nodules) 4(>12 nodules) or CC-like 1 (1–3 nodules) 2 (4–7 nodules) 3 (8–11 nodules) 4 (>12 nodules).

### Immunoblotting

Cell line and liver tissue extracts were lysed in the lysis buffer (25 mM 4-(2-hydroxyethyl)-1-piperazineethanesulfonic acid (HEPES), 400 mM NaCl, 1.5 mM MgCl2, 0.2 mM EDTA, 1% NP40, protease inhibitor and phosphatase inhibitor and normalised before western blot analysis was performed using antibodies listed in online supplementary material.

### Nanostring and PCR analysis

RNA extracted from livers and cells were subjected to reverse transcription and subsequently underwent quantitative PCR with the use of the Applied Biosystems Inc (ABI) 7300 real-time PCR system (Carlsbad, California). The following ABI primers (ABI identifier) were used: LTβR (Mm00440235_m1) and Gapdh (Mm99999915_g1). Analysis was performed according to ABI's manufacturer's protocol with target genes normalised to endogenous levels of GAPDH and 2(−ΔΔC(T)) method used as previously described.^18^ Additional methods are listed in online supplementary material.

### Hepatic cell lines and in vitro experiments

Human HCC cell lines HepG2, Huh1, HLE, Huh7 and human cholangiocellular cell lines Oz, KMBC, HuCCT1 and Mz-CHA-1 were generously provided by Dr Xin Wei Wang, National Cancer Institute, Bethesda, Maryland. Additional methods are listed in online supplementary material.

### Flow Cytometry

LTβR expression levels were determined using PE-labeled anti-LTβR (1:40) BD Pharmagen (Stamford, Connecticut, USA) with analysis performed by Becton Dickenson Canto cytometer. In vitro stimulation experiments were performed as indicated using agonistic goat antihuman LTβR.

### Microarray analysis

The Llovet data set (ICC=143, normal biliary epithelial cells=6) was extracted from Geo Omibus (GSE32225).^19^ The mean was calculated for each gene symbol and log 2-transformed using R script (V.3.0.1). Log-transformed data were then imported into Biometric Research Branch (BRB) Array V.4.3.2. Additional methods are listed in online supplementary material.

### Statistical analysis

Differences between groups were compared using either Mann-Whitney U test or two-tailed unpaired Student's t test. Survival differences were compared using Log-rank (Mantel-Cox) test. Statistical analysis was performed using GraphPad Prism 6.0 (La Jolla, California, USA). p values <0.05 were considered statistically signiﬁcant.

## Results

### LTβR signalling is critical for AKT/CAT-initiated hepatic tumour proliferation and progression

**Figure 1:**
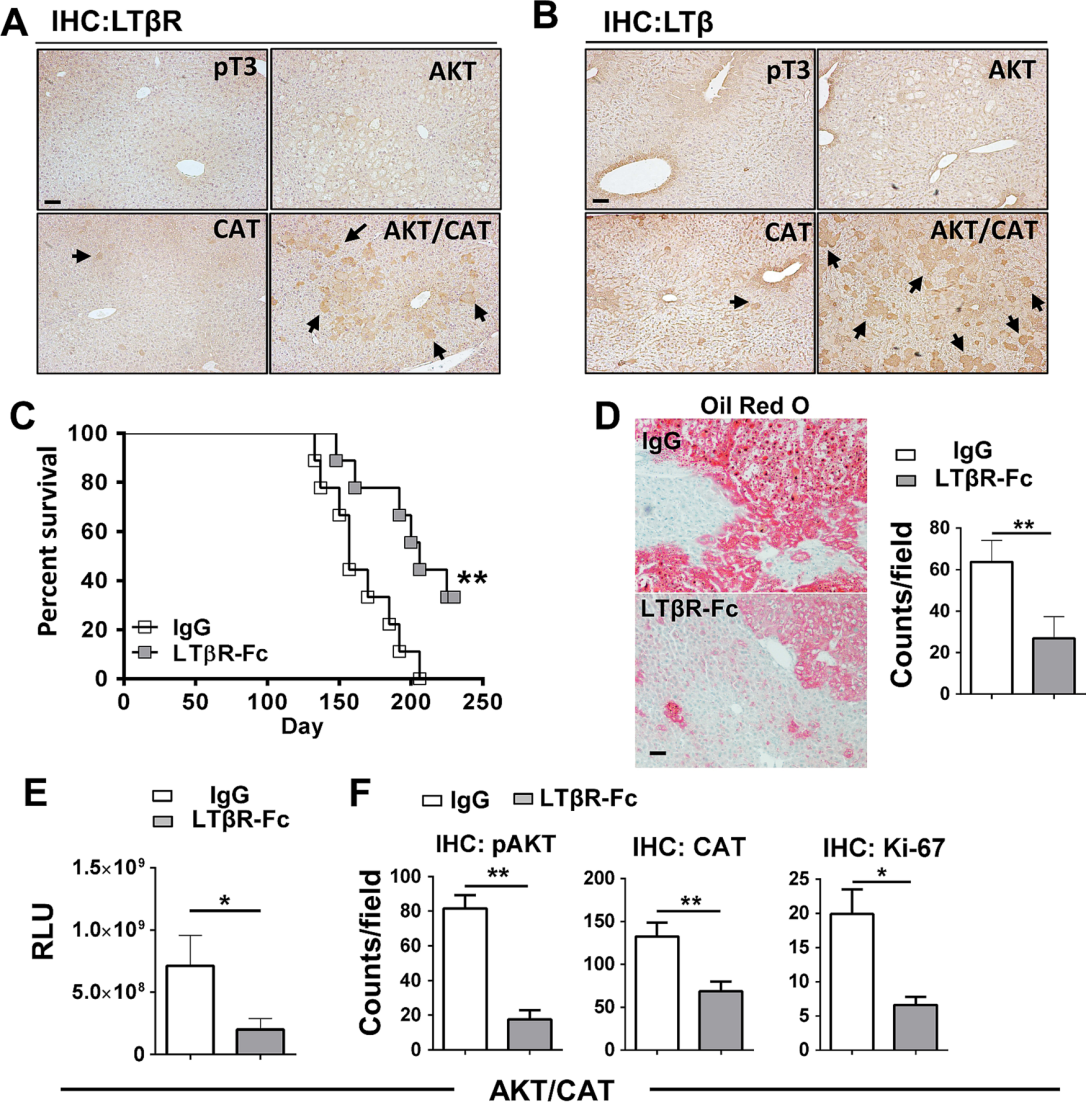
Upregulated LTβ/LTβR expression is critical for AKT/CAT-initiated tumour progression. Immunohistochemical staining (IHC) of livers harvested day 40, post injection with pT3, AKT, CAT or AKT/CAT with antibodies against LTβR (A) and LTβ (B). Arrows denote representative areas of positive staining. (C–F), LTβR-Fc (100 μg) or control IgG (100 μg) were administered twice/week in AKT/CAT-injected mice starting day 10, post oncogene injection, and continued for 8 weeks (C–E) or 4 weeks (F). (C) Survival curves were analysed and data is representative of two independent experiments (IgG and LTβR-Fc; n=9). (D) Representative Oil Red O stains of moribund livers with quantitation. (E) Tumour progression in moribund mice was analysed by measuring serum levels of cotransfected reporter Gaussia luciferase (relative luciferase units, RLU). (F) Quantification of IHC staining for pAKT, CAT and the proliferation marker Ki-67 in the livers day 40 post AKT/CAT injections. (D and F), positive cell numbers (counts) in at least 10 non-lapping fields (n=3–5 mice/group) were determined using cell profiler quantitation software. Bars represent mean values ±SEM. *p<0.05; **p<0.01. Scale bars, 100 µm. CAT, catenin.

To investigate the role of the lymphotoxin signalling following oncogenic activation in the liver, C57BL6/J mice were hydrodynamically transfected with the combination of AKT/CAT or empty vector (pT3). IHC staining for LTβR and LTβ of liver sections obtained 40 days after transfection of AKT/CAT showed increased levels of staining when compared with single oncogenes (figure 1A, B), with expression primarily restricted to regions of hepatic dysplasia. Isolated liver RNA obtained 40 days after AKT/CAT transfection displayed a modest increase in LTβ, while in contrast, LTβR ligand LIGHT (TNFSF14) and the LTα mRNA levels were not significantly changed relative to empty vector (pT3) control (see online supplementary figure S1A). Quantitative PCR revealed a mean 1.8-fold increase in LTβR in AKT/CAT-initiated tumours relative to pT3 transfected control livers (see online supplementary figure S1B). IHC staining for LTβR revealed increased expression by day 14 which intensified with the presence of tumour at day 49 and day 85 (see online supplementary figure S1C). LTβR ligand competitive antagonism was evaluated using a soluble form of the LTβ receptor (LTβR-Fc). Administration was initiated day 10 post oncogene transfection to circumvent potential complications associated with hydrodynamic injection-induced acute liver injury^20^ and/or oncogene integration,^13^ and continued for 8 weeks. LTβR-Fc significantly extended median survival to 206 days versus 157 days in Ig-treated control mice (figure 1C). A distinguishing feature of AKT/CAT-initiated oncogenesis is lipid accumulation,^14^ which is also associated with chronic LTβR activation.^12^ Treatment with LTβR-Fc decreased lipid accumulation as detected by Oil red O staining for neutral fats and quantitative microscopy (figure 1D). Moreover, prolonged administration of LTβR-Fc significantly reduced mean serum levels of the cotransfected oncogene reporter Gaussia luciferase by threefold (figure 1E) confirming a reduction in tumour burden. Quantitative analysis of liver IHC staining for pAKT_ser473_, CAT and the proliferation marker Ki-67 demonstrated significantly reduced levels of oncogenes and the number of proliferating hepatocytes (figure 1F), but failed to significantly alter serum liver aspartate transaminase (AST)/alanine transaminase (ALT) enzyme and total bilirubin levels (see online supplementary figure S2A). There was no significant change in tumour morphology by Fc-treatment (see online supplementary figure S2B). AKT/CAT-transformation of hepatocytes and subsequent LTβ/LTβR upregulation are thus implicated in tumour proliferation and progression.

### LTβR agonism enhances the proliferation, progression and prevalence of lipogenic and ICC-like tumours

**Figure 2:**
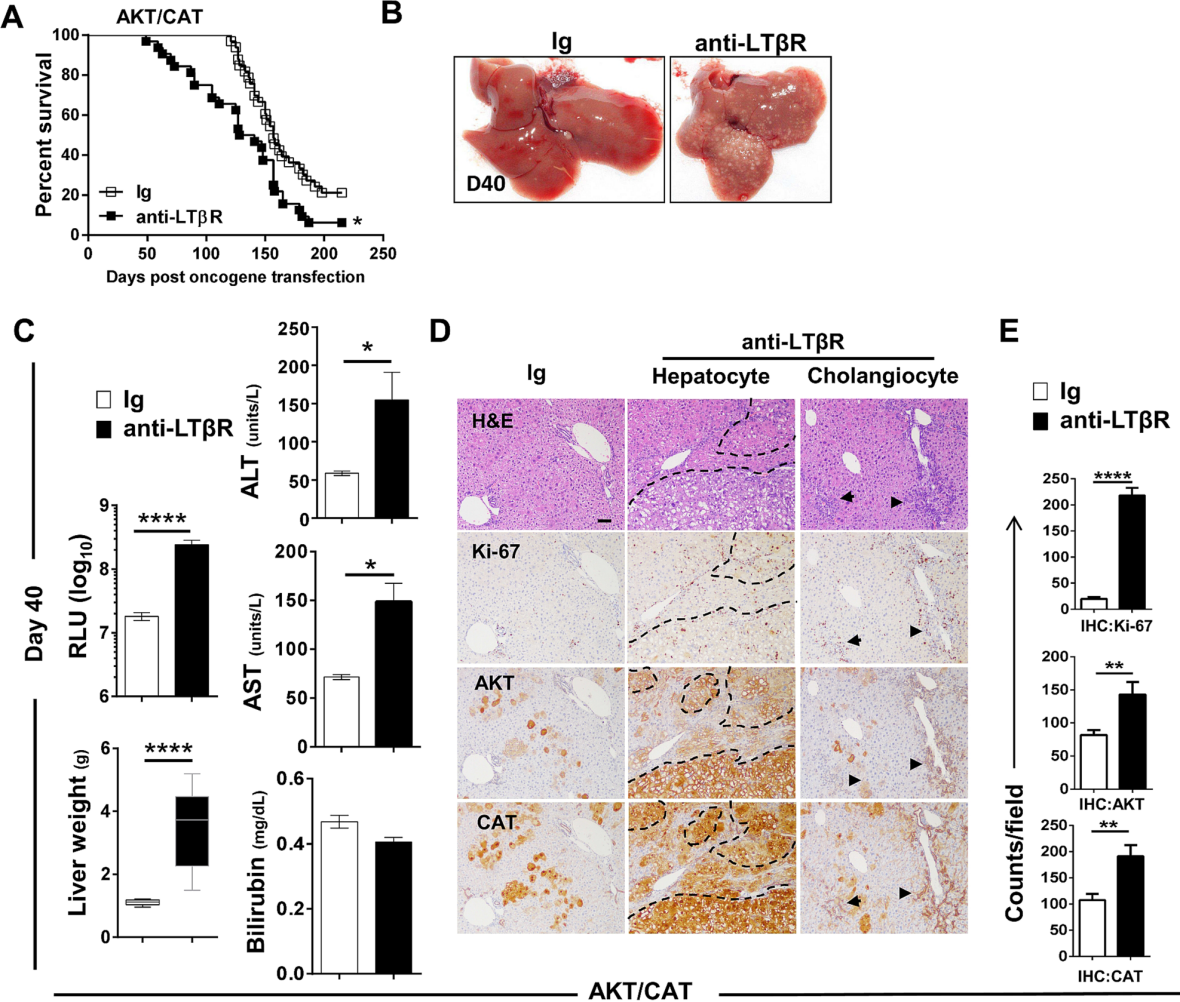
Chronic activation of the LTβR significantly augments AKT/CAT-initiated tumour progression and proliferation. **(**A–D) 100 μg agonistic antibody LTβR clone 4H8 (anti-LTβR) or Ig control were administered twice/week in AKT/CAT-injected mice starting day 10, post oncogene injection, and continued for 8 weeks (A) or 4 weeks (B–D). (A) Four independent experiments (Ig, n=32 anti-LTβR, n=33) were combined and survival was analysed. (B–D), Histology and pathology analysis of livers harvested at day 40 post AKT/CAT injections following anti-LTβR or Ig control treatments. (B) Representative macroscopic images of the livers were shown. (C) Liver weight and serum levels of cotransfected reporter Gaussia luciferase (relative luciferase units, RLU) (Ig (n=20), anti-LTβR (n=18), aspartate transaminase (AST) (units/L), alanine transaminase (ALT) (units/L) and total bilirubin (mg/dL (n=7/group)). (D) Representative H&E and IHC staining for Ki-67, pAKT and CAT. Dashed lines outline regions of tumour. Arrows illustrate area of increased cholangiocyte proliferation/dysplasia. Scale bar, 100 µm. (E) Positive cell numbers were quantitated using at least 11 non-lapping fields (n=3–6 mice/group). Bars represent mean values±SEM. *p<0.05, **p<0.01, ****p<0.0001. CAT, catenin; IHC, immunohistochemical.

**Figure 3:**
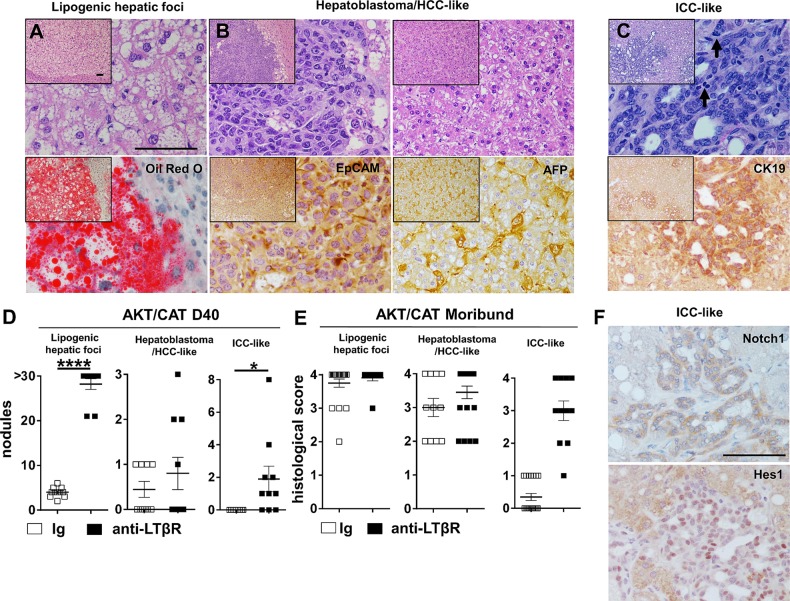
Effect of LTβR overactivation on spectrum of tumour types present in AKT/CAT-initiated tumours. (A–C) Representative H&E staining (×400 with insets ×100) of morphologies that define tumour types as observed in AKT/CAT and AKT/CAT/LTβR initiated livers. (A) Lipogenic hepatic foci were large lipid dense hepatocytes displaying clear cell morphology which stain positive for Oil Red O. (B) Hepatoblastoma/HCC-like nodules displaying multiple characteristics, most commonly small cell, undifferentiated subtypes staining positive for EpCAM. (C) CC-like nodules/regions that form ductular/pseudoglandular patterns with mitotic figures (arrows) and staining for CK19. (D–E) Livers with distinct nodules were counted (D) with histological scoring performed to assess severity for livers with coalescing lesions (E). Histological evaluation of H&E stains from day 40 (D) and moribund (E) AKT/CAT-transfected livers following 4 weeks (D) or 8 weeks (E) of anti-LTβR (n=10 (D40), n=11 (moribund)), LTβR-Fc (n=8 (D40)) or Ig (n=10 (D40), n=20 (moribund)) treatment was performed. (E) Histological scoring (described in methods) was performed. Mann-Whitney U test was used to determine significance. *p<0.05, ****p<0.0001. (F) Representative images of IHC stained intrahepatic cholangiocarcinoma (ICC)-like structures from AKT/CAT/LTβR moribund livers was performed using antibodies against Notch1 and Hes1. CAT, catenin; HCC, hepatocellular carcinoma; IHC, immunohistochemical.

Malignant transformation of hepatic adenoma to carcinoma is frequently accompanied by mutation of the β-catenin pathway.^21^ To investigate the ability of LTβR activation to drive AKT/CAT aggressiveness, we administered agonistic anti-LTβR mAb, clone 4H8 (anti-LTβR) for either 4 weeks or 8 weeks beginning at day 10 post transfection of C57/BL6 mice. Survival was significantly reduced with chronic anti-LTβR treatment (figure 2A), and dramatically different tumour frequencies were observed between livers from control and anti-LTβR-treated mice at day 40 (figure 2B). A 13.3-fold increase in the serum level of cotransfected luciferase was detected in AKT/CAT/anti-LTβR mice (2.4×10^8^ relative luciferase units, RLU) compared with the Ig control (1.8×10^7^ RLU) at day 40 (figure 2C), which was consistent with the threefold increase in liver weights at day 40, (Ig=1.01(g) vs anti-LTβR=3.4 g) (figure 2C). Additionally, serum liver enzymes ALT and AST were significantly elevated at day 40 following anti-LTβR treatment (figure 2C). Increased hepatocyte and cholangiocyte proliferation were also observed in AKT/CAT/anti-LTβR livers at day 40 as detected by IHC staining for the proliferation marker Ki-67 (figure 2D), with multiple coalescing proliferative regions of hepatic dysplasia with cellular atypia observed in hepatocellular and bile duct regions. Consistently, increased frequency of IHC staining was observed for AKT and CAT oncogenes that localised together and in areas of cellular dysplasia, reflecting increased tumour burden (figure 2D). Quantitative analysis of IHC confirmed significant increases in AKT and CAT, as well as Ki-67 expression in AKT/CAT/anti-LTβR livers (figure 2E). These data suggest that LTβR-activation accelerates AKT/CAT-initiated tumour formation and progression. It should be noted that in the absence of AKT/CAT oncogene expression, anti-LTβR failed to significantly alter proliferation, endogenous oncogene expression (see online supplementary figure S3A), and serum liver enzyme levels (see online supplementary figure S3B). We next focused our analysis on morphological characteristics of liver tumours harvested at day 40 or from moribund mice subsequent to chronic activation of LTβR. AKT/CAT-initiated tumours display multiple pathologies.^14^ Therefore, we further characterised nodules based on several morphological and molecular characteristics. Specifically, lipogenic hepatic foci are comprised largely of lipid fluid hepatocytes displaying clear cell morphology, as defined by with Oil Red O ere(figure 3A) and AFP negative staining (see online supplementary figure S4). Hepatoblastoma/HCC-like nodules display a diverse morphological continuum of predominately epithelial small cell, undifferentiated subtypes^22^ (figure 3B) with trabecular and cholangioblastic features including regions of desmoplasia (see online supplementary figure S5B (arrow)) which are present as tumours progress. These nodules stain positive for the hepatic stem/progenitor cell markers, Epithelial cell adhesion molecule (EpCAM) (figure 3B) and Glypican-3 with sporadic AFP staining (see online supplementary figure S4). Furthermore, CD34 a potential marker for detecting HCC/ICC cancer stem cells^23^ and vascular endothelial cells,^24^ was also increased following LTβR triggering (see online supplementary figure S4). ICC-like lesions form diffuse, ductular/pseudoglandular patterns with the appearance of mitotic figures (figure 3C arrows) with ICC-like nodules staining positive for CK8 (a marker of preneoplastic hepatic lesions^15^ ^25^), cholangiocyte marker CK19 (figure 3C), Ki-67, and Masson's trichrome (fibrosis) (see online supplementary figure S4). Moreover, transposon expression, pAKT and β-cat IHC staining was observed in AKT/CAT-associated morphologies (see online supplementary figure S4). Histological evaluation of H&E stains from day 40 and moribund AKT/CAT-transfected livers following 4 weeks or 8 weeks of anti-LTβR or Ig treatment was performed (figure 3D, E). Agonist anti-LTβR treatment aggressively increased tumour burden (figure 2) with significant increases in lipogenic foci and ICC-like nodules observed at day 40 (figure 3D). Given the coalescing nature of the resulting tumours and sheer numbers of nodules in moribund livers, histological scoring was performed as described in Methods. Moribund livers following 8 weeks of anti-LTβR or Ig treatment displayed a preponderance of lipogenic foci with mean histological scores of 3.7 for anti-LTβR and 3.5 for Ig (figure 3E) and similar incidence of hepatoblastoma/HCC-like tumours following agonism (figure 3E). In contrast, anti-LTβR livers were interspersed with regions of ICC-like lesions, with mean histological scores of 0.35–3.0 in Ig and anti-LTβR livers, respectively. Furthermore, ICC mediator Notch1 and its downstream target Hes1^26^ were exclusively detected in AKT/CAT ICC-like lesions (figure 3F).

### LTβR activation rapidly accelerates AKT/NICD-initiated ICC

**Figure 4:**
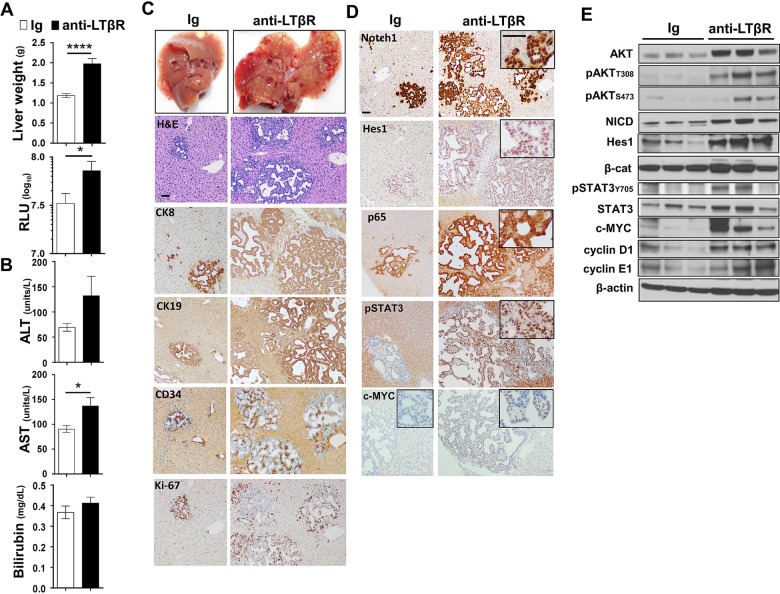
LTβR rapidly accelerates AKT/NICD-initiated intrahepatic cholangiocarcinoma (ICC) development. (A–E) Mice were hydrodynamically injected with AKT/NICD to initiate ICC formation, and then treated with anti-LTβR or Ig for 4 weeks starting on day 10, post oncogene delivery. Livers and serum were analysed at day 40 post AKT/NICD injection. (A) Liver weights (g) (n=19/group), serum Guassia Luciferase (relative luciferase units, RLU) (n=24/group) (B) aspartate transaminase (AST)/alanine transaminase (ALT) (units/L) and total bilirubin levels (n=9–10/group) were measured. (C) Representative images comparing multifocal tumours, as well as H&E and IHC staining for CK8, CK19, Ki-67, CD34 was performed. (D) Representative IHC staining using antibodies against pAKT, Notch1, Hes1, pSTAT3, NFκBp65 and c-MYC. Scale bar, 100 µm. (E) Western blot (WB) analysis for indicated markers was performed with liver tissue lysates from three representative mice per group. All scale bars represent mean values±SEM. Mann-Whitney U test used to determine significance, *p<0.05 ****p<0.0001. CAT, catenin; NICD, Notch-intracellular domain; IHC, immunohistochemical.

Based on the results described above, a role for LTβR signalling in the progression of ICC was further investigated using a recently described ICC model driven by oncogenes AKT and active form of Notch, NICD.^15^ Hydrodynamic transfection of AKT/NICD, combined with chronic administration of anti-LTβR, dramatically increased liver weight (VC=1.1 g to anti-LTβR=2.2 g) and levels of cotransfected oncogenic reporter, Gaussia luciferase increased twofold (figure 4A). AST serum levels were significantly elevated at day 40 (figure 4B). Gross examination of these livers suggests LTβR-activation rapidly accelerates progression and pathogenesis of ICC (figure 4C). H&E staining of AKT/NICD ICC nodules suggests well defined foci with a ductular/pseudoglandular morphology (figure 4C) and the frequent appearance of mitotic figures (see online supplementary figure S6 arrows). IHC staining revealed increased expression of CK8, CK19, CD34 and Ki-67 in anti-LTβR treated livers (figure 4C). Likewise, anti-LTβR livers displayed increased staining of transfected Notch1, mediator Hes1 and oncogenic drivers NF-κB p65, pSTAT3 and c-MYC observed in ICC nodules (figure 4D). LTβR-accelerated tumour burden was further documented by increased levels of the transfected oncogenes NICD, with increased levels of AKT, activated pAKT_Thr308_, pAKT_Ser473_, NICD and Hes1 observed at day 40 (figure 4E) by western blot with liver lysates derived from AKT/NICD/anti-LTβR treated mice. To understand the mechanisms of LTβR-facilitated ICC progression, we next examined whether LTβR agonism indirectly activates other functionally validated liver cancer pathways.^3^ Increased levels of CAT, c-MYC, and pSTAT3_Tyr705_ were detected in AKT/NICD/anti-LTβR livers (figure 4E). Furthermore, cyclinD1 and E1, recently shown to regulate ICC through interaction with p27,^26–28^ were elevated following LTβR activation (figure 4E). To corroborate AKT/NICD-related molecular findings, IHC staining was performed on AKT/CAT-initiated tumours, with NF-κB p65 selectively expressed in ICC-like lesions (see online supplementary figure S7A). Furthermore, in the AKT/CAT and AKT/NICD models, a significant increase in c-MYC transcription was observed following LTβR agonism (see online supplementary figure S7B).

### LTβR agonism preferentially enhances AKT-initiated hepatic tumour development and reduces survival

**Figure 5:**
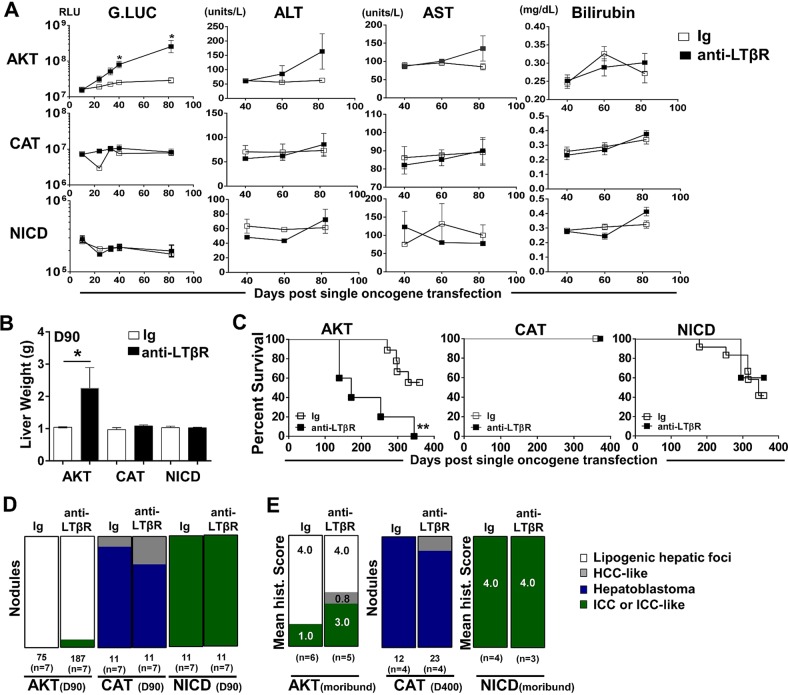
LTβR agonism preferentially promotes AKT-initiated tumour development. (A–E) 100 μg of anti-LTβR or Ig were administered twice/week in single oncogene CAT, AKT or NCID injected mice, starting day 10 post oncogene injection and continued for 8 weeks. (A) Serum analysis of Guassia Luciferase (RLU), aspartate transaminase (AST)/alanine transaminase (ALT) (units/L) and total bilirubin (mg/dL) levels were measured at indicted time points. (B–C) Liver weights (g) at day 90 (B) and per cent survival were determined (C). (D–E) Nodules were counted or histologically scored using H&E stained liver sections from (D) Day 90 from AKT/Ig (n=7) AKT/anti-LTβR (n=7), CAT/Ig (n=7) CAT/anti-LTβR (n=7) and NICD/Ig (n=7) NICD/anti-LTβR (n=7). (E) Mean histological scoring (1 mild to 4 severe) was performed from H&E stained moribund or Day 400 liver sections from AKT/Ig (n=6) AKT/anti-LTβR (n=6) and NICD/Ig (n=4) NICD anti-LTβR (n=3) with nodule counts performed from CAT/Ig (n=4) CAT/anti-LTβR (n=4) livers. All scale bars represent mean values±SEM. Mann-Whitney or log-rank tests were performed. *p<0.05, **p<0.01. CAT, catenin; NICD, Notch-intracellular domain.

We next investigated the ability of LTβR signalling to modulate hepatic tumour progression in models driven by only AKT,^29^ CAT^30^ ^31^ or NICD.^26^ Sequential serum analyses of Guassia Luciferase, AST, ALT and total bilirubin levels suggest that anti-LTβR treatments selectively promote AKT-initiated tumour progression (figure 5A), which was consistent with significantly increased liver weights at day 90 (figure 5B) and survival observed in AKT/anti-LTβR-treated mice (figure 5C). Day 90 histological evaluation of tumour nodules supports direct collaboration between AKT and LTβR signalling, averaging 11.4 (Ig, n=7) vs 29.4 (anti-LTβR, n=7) nodules/liver following 8 weeks of treatment. In contrast, tumour burden in CAT/anti-LTβR and NICD/anti-LTβR-treated mice was unchanged relative to Ig control (figure 5C, D). Similar to AKT/CAT/anti-LTβR, AKT and AKT/anti-LTβR tumours were predominantly lipogenic hepatic foci, with LTβR agonism inducing the appearance of ICC-like nodules/regions by day 90 (figure 5B) and increasing frequency in moribund mice. In these mice, the mean histological score increased from 1.0 (Ig, n=6) to 3.0 (anti-LTβR, n=5) (figure 5E). Transfection of CAT results in the formation of distinct hepatoblastoma and HCC-like nodules at day 90 and day 400 (figure 5D, E), while NICD transfected livers were similar in morphology to AKT/NICD-initiated livers; solely ICC at day 90 and time of morbidity (Fig 5D, E). Cholangiocyte proliferation/dysplasia was defined by appearance of cholangiocyte proliferation, biliary dysplasia and/or bridging, with the lack of well defined glandular patterns. Noteworthy changes were observed in biliary proliferation (H&E) following AKT or AKT/CAT/anti-LTβR, but not observed in pT3 or CAT/anti-LTβR livers at day 40 following 4 weeks of treatment (see online supplementary figure S8A, arrows). Furthermore, histological scoring of day 40 and moribund H&E stained liver sections suggests AKT/CAT/anti-LTβR mediated cholangiocyte dysplasia (see online supplementary figure S8B). Therefore, LTβR signalling preferentially enhances AKT-initiated progression with the concomitant activation with CAT further enhancing the appearance of ICC-like morphology.

### LTβR signalling is involved in human ICC pathogenesis

**Figure 6:**
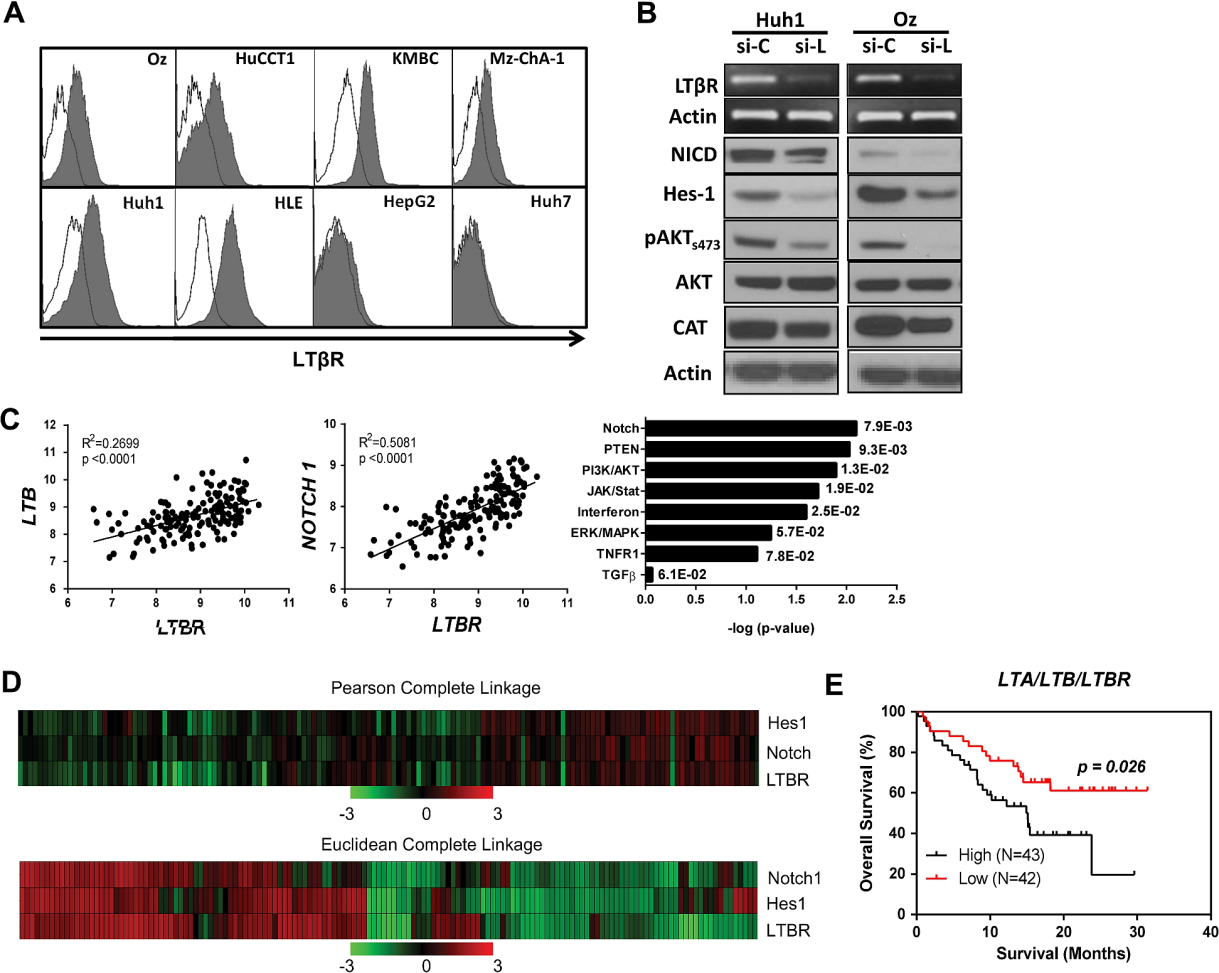
LTβR signalling regulates oncogene activities in human liver cancers and correlates with poor survival of patients with intrahepatic cholangiocarcinoma (ICC). (A) Flow cytometry analysis of LTβR surface expression in cholangiocarcinoma (Oz, HuCCT1, KMBC, MZ-ChA-1) and HCC (Huh1, HLE, HepG2, Huh7) cell lines. (B) siRNA specific for LTβR (si-L) was transfected into Huh1 and Oz cells. 48 h later, LTβR shutdown efficiency was confirmed by RT-PCR (top two panels), and Notch1/Hes1/AKT/pAKT_s473_/CAT protein level were analysed by western blot (WB) with cell lysates (bottom panels) compared with control siRNA (si-C) (C) Reanalysis of Lovelt ICC cohort, LTβR expression relative correlation with LTβ (left panel) and Notch1(centre panel) expression. Further, IPA of differentially expressed genes of ICC versus normal was performed to detect signalling pathways associated with high *LTBR* gene expression (right panel). p Values on the side of the bars are from Fisher's exact test using the IPA database, X-axis is–log p—value of IPA calculated p value. (D) LTβR, Hes1 and Notch1 heat map was constructed from statistically significant ‘proliferative class’ genes between Lovelt cohort ICC versus normal biliary epithelial cells (494 genes), normalised log2 transformed and hierarchal clustering performed (Pearson's correlation-complete linkage). (E) Patient survival data from TIGER-LC Thailand cohort was stratified based on LTβR high (n=43) and low (n=42) expressions. CAT, catenin; HCC, hepatocellular carcinoma; IPA, ingenuity pathway analysis.

To confirm the novel role of LTβR signalling in the pathogenesis of human liver cancers, particularly ICC, we first screened by flow cytometry several human liver tumour cell lines for LTβR expression. We found that LTβR was widely expressed by all four cholangiocarcinoma cell lines (Oz, KMBC, HuCCT1 and Mz-ChA-1) we tested, as well as by two HCC cell lines Huh1 and HLE (figure 6A). Knockdown of LTβR in Huh1 and Oz cells with targeting siRNA resulted in decreased protein expression/activation of pAKTser473, CAT, NICD and Hes1 levels at 48 h post transfection (figure 6B), suggesting that LTβR signalling may be important for maintaining the activity of these oncogenes in human liver cancer cells. Since recent human ICC transcriptome analysis revealed elevated LTβR expression in a molecularly defined proliferative subtype of patients with ICC,^19^ we then performed further transcriptome analysis on this cohort which revealed *LTBR* expression was correlated with expression of *LTB* (R^2^=0.2699) and *NOTCH1* (R^2^=0.5081) (figure 6C). Moreover, ingenuity pathway analysis of differentially expressed genes of ICC vs normal were enriched in Notch, phosphatase and tensin homolog (PTEN) and PI3K/AKT signalling pathways and associated with high *LTBR* gene expression (figure 6C, right panel). In addition, hierarchal clustering of ‘proliferative class’ genes revealed a subset of significantly regulated ‘proliferative’ genes similarly clustering with *LTBR*, *NOTCH1* and *Hes1* (figure 6D). Furthermore, an ICC cohort of Thai patients obtained for study through the TIGER-LC consortium (Chaisaingmongkol *et al*, manuscript in preparation) stratified LTβR high (n=43) expression with significantly worse survival as compared with ICC cases with LTβR low (n=42) expression (figure 6E). Subsequent analysis of tissue samples from human ICCs revealed varying morphological patterns with positive staining observed for LTβ, LTβR, β-cat (membrane and nuclear), pAKT and Hes1 (see online supplementary figure S9). LTβR and LTβ positive cells with ICC and leucocyte morphology (see online supplementary figure S9, arrows) were observed. Together, these results suggest a link between the LTβR pathway and functionally validated drivers of ICC that strongly associate with human ICC.

## Discussion

These results reveal the novel interplay between the LTβ/LTβR inflammatory pathway and key oncogenes that drive liver malignancy, particularly lipogenic hepatic foci and ICC-like lesions. We provide evidence that AKT/CAT combined activation can mediate the upregulation of LTβ/LTβR expression and further demonstrate LTβR signalling is a central activator during tumour development. Moreover, prolonged LTβR activation significantly enhanced proliferation, skewing AKT/CAT-induced tumour morphology towards the appearance of ICC-like lesions and accelerating AKT/NICD-initiated ICC. Intriguingly, LTβR-mediated tumour progression was largely dependent on oncogenic AKT signalling as LTβR agonism failed to alter survival in single oncogene CAT or NICD-initiated tumour models. The LTβR is broadly expressed in human liver cancer cell lines and contributes to maintaining AKT activation and the accumulation of NICD. Transcriptome profiling of ICC cohorts confirmed a significant relationship between LTβR, NOTCH and AKT/PI3K signalling pathways. Further, poor survival of patients with ICC significantly correlated with higher LTβR network expression.

Defining the mechanisms underlying LTβ and/or LTβR upregulation during malignancy has been elusive. Simonin *et al*^32^ recently elucidated a HCV-mediated mechanism that directly regulates tumour-specific increases in LTβ, independent of the oncogenic driver, N-MYC. We demonstrate specific concomitant oncogenic activation upregulates LTβ and LTβR expression in vivo. Similarly, we detected increased LTβ expression in AKT/NICD and cMET/CAT-hydrodynamically transfected livers (see online supplementary figure S10A, B). It is conceivable, oncogenic activation is intrinsically regulating LTβ induction due to immense transcriptional amplification following transformation, or mediated from extrinsic factors in the tumour milieu.

It is well established that liver cancer emerges following many years of chronic liver damage and compensatory hepatic cell proliferation. Interestingly, combined AKT/CAT or AKT/NICD activation was required for robust LTβR-mediated proliferation since hydrodynamic transfection of CAT or NICD alone failed to markedly enhance proliferation. We speculate the LTβR-mediated enhancement of proliferation is augmenting progression and skewing the pathological appearance of AKT/CAT-associated tumour phenotypes, such as ICC-like lesions. Based on work from Sia *et al*,^19^ who molecularly defined ‘inflammatory’ and ‘proliferative’ subclasses of human ICC tumours with higher LTβR expression (2.3-fold) associating with ‘proliferative’ ICC subclass and worse survival, it is reasonable to conclude LTβ or LTβR expression could serve as a marker for proliferating ICC. The appearance of cholangiocellular lesions in AKT-transfected livers has been described.^33^ We expanded on this observation and demonstrate that LTβR agonism+AKT and to a greater extent LTβR agonism + AKT/CAT, results in tumour pathology characterised by lipogenic hepatic foci with interspersed regions of ICC.

Recent studies have defined Notch1 as an essential regulator of liver progenitor cell fate and critical for the development of ICC.^15^ ^26^ Moreover, IHC staining for AKT, Notch and CAT suggests ICC-like regions have elevated expression levels compared with adjacent hepatic foci. It is plausible that upregulation by either transposon or endogenous AKT, Notch and CAT oncogene in cholangiocytes or liver progenitor cells initiates biliary tumour formation or hepatocyte dedifferentiation following malignant transformation as recently described.^34^ Furthermore, we provide evidence that LTβR signalling is also important in maintaining endogenous Notch1-ICD/Hes1 in human liver cancer cell lines and correlates with Notch1 expression/signalling in patients with ICC. Liu *et al*^35^ demonstrated that TNF, another TNF-superfamily member and subsequent IKKα accumulation in HCC cell lines were capable of driving proliferative advantage via Notch1-FOXA2 suppression. Additionally, evidence identifies TNF-mediated Notch signalling in the progression of pancreatic cancer.^36^ Canonical NF-κB signalling has been reported to be critical for AKT oncogenicity.^37^ Therefore, it is possible that LTβR-NF-κB regulation of AKT with subsequent activation of NICD could facilitate tumour formation. Using the AKT/NICD ICC model,^15^ we demonstrate additional LTβR-specific molecular changes occur in association with upregulation of c-MYC,^38^ cyclin D1, cyclin E1^39^ and CAT.^40^ ^41^ It remains uncertain if these oncogenic proteins are directly regulated by LTβR signalling or indirectly induced by factors in the microenvironment. Regardless, accumulations of these well established oncogenic drivers are capable of driving tumour development.

Two emerging hallmarks of cancer are deregulated metabolism and chronic inflammation.^42^ AKT/CAT-initiated livers are lipid dense, similar to those resulting from overactivated AKT which induces metabolic dysregulation, including a hypoglycaemic, hypoinsulinaemic and hypertriglyceridaemic phenotype with fatty liver and hepatomegaly.^29^ Moreover, hepatocyte LTβR signalling has also been reported to regulate metabolic function, lipid homoeostasis,^12^ and recently the transition from non-alcoholic steatohepatitis to initiated HCC.^43^ It is therefore intriguing to speculate that the reduction in AKT/CAT-initiated tumour development by LTβR-Fc is mediated in part through a normalisation of metabolic functions.

Chronic inflammation is an established risk factor and pathological marker for biliary tract cancers.^44–46^ LTβR-mediated oncogene activation failed to significantly enhance proliferation in human tumour lines, indicating the robust hepatocyte and cholangiocyte cell proliferation observed following LTβR agonism could in part result from increased microenvironmental factors. LTβR signalling is well known to recruit lymphoid and myeloid cells into lymphoid organs and tumours,^47^ ^48^ and activation mediates accumulation of macrophages and NKT/T cells in association with increased expression of CXCL10 and CCL2 in AKT/CAT and AKT/NICD tumours (online supplementary figure 11 A, B). CXCL10 is expressed by hepatocytes during chronic viral hepatitis,^49^ induced by LTβR activation via NF-κB and is considered as one of the main chemoattractants for tumour-infiltrating immune cells. Furthermore, Dubois-Pot-Schneider *et al*^50^ provide molecular evidence that TNF, IL-6 and TGF-β-related signatures are increased during dedifferentiation of tumour-derived hepatocyte-like cells to progenitor cells. It remains unclear if LTβR mediated chemokine recruitment of inflammatory cells, and subsequent activation of NF-κB p65 and pSTAT3 observed in livers from AKT/CAT and AKT/NICD-transfected mice is promoting hepatocyte dedifferentiation or ICC-like formation. However, given the inflammatory aetiology of cholangiocarcinoma, the investigation of inflammation mediated through the LTβR, and its possible collaboration with molecular events to alter cell fate in the liver, may prove a rich avenue for further study. Collectively our results linking LTβR signalling and oncogenic activation suggest that drugs targeting LTβR signalling combined with AKT or Notch inhibitors may have important clinical implications.

## Supplementary Material

Web supplement
