# Supplementary material for: LTβR signalling preferentially accelerates oncogenic AKT-initiated liver tumours
Source: Gut. 2015 Jul 23;65(10):1765–75. doi: 10.1136/gutjnl-2014-308810 (PMC5036232; doi:10.1136/gutjnl-2014-308810)
Supplement: Web supplement [file gutjnl-2014-308810-s1.pdf]

Supplemental Methods

Mice

C57BL/6 mice were originally purchased from either The Jackson Laboratories (Jax) (Bar Harbor, MA) or Charles River (NcR) (Frederick, MD) and were bred and maintained in specific pathogen-free (SPF) housing. Experiments were performed with appropriate littermate or strain and age-matched mice. Mice were humanely euthanized when moribund in accordance with Frederick National Laboratory Institutional Animal Use and Care protocol.

Serum assays

The Histology and Tissue Core Facility at the Frederick National Laboratory for Cancer Research routinely performed serum enzyme assays for Aspartate aminotransferase (AST), Alanine aminotransferase (ALT), and total bilirubin.

Immunohistochemsitry

Immunohistochemical staining for LTβR, LTβ, AFP, CK19, CK8, Notch1, Hes1, pAKT, Glypican-3, and c-MYC was performed on paraffin sections using the Ultravision LP detection system (Thermo Fisher Scientific, Fremont, CA), according to the manufacturer’s instructions. Quantitation of positive nuclei or cells was performed using optimized cell profiler (Cambridge, MA) pipelines from at least 11 non-overlapping fields (n=3-6 mice). ICC tissue microarray (LV642) was purchased from US biomax, Inc. (Rockville,MD) and stained with antibodies against LTβ, LTβR, pAKT, Hes-1, and β-cat.

Immunohistochemical Antibodies

Antibody	Dilution	Manufacturer	Cat #
Ki-67	1:200	Abcam	Ab15580
pAKT S473	1:50	Cell Signaling	3787
β-catenin	1:100	Cell Signaling	9562
CK8 (TROMA-I)	1:100	DSHB	
CK19	1:100	Abcam	ab15463
CK19	1:500	Abbomax	602-670
Notch1	1:100	Cell Signaling	3608
Hes1	1:500	Cell Signaling	11988
CD34	1:50	eBioscience	14-0341-85
pSTAT3 Tyr705	1:100	Cell Signaling	9145
p65	1:200	Cell Signaling	8242
c-MYC	1:50	Abcam	ab32072
Glypican-3	1:100	Abcam	Ab66596
LTβR	1:100	Carl Ware	Clone 4H8
Human LTβR	1:100	Abcam	Ab70063
AFP	1:100	Dako	A0008
EpCAM	1:200	Novus	NBP2-27361
LTβ	1:100	Abcam	Ab64835

Western Blot antibodies

Antibody	Dilution	Manufacturer	Cat #
AKT	1:1000	Cell Signaling	9272
pAKT Ser473	1:1000	Cell Signaling	4058
pAKT Thr308	1:1000	Cell Signaling	9275
Notch1	1:1000	Cell Signaling	3608

Hes1	1:1000	Cell Signaling	11988
β-catenin	1:200	Abcam	2365
pStat3 Tyr705	1:1000	Cell Signaling	9131
STAT3	1:1000	Cell Signaling	9132
c-Myc	1:200	Santa Cruz	sc-764
Cyclin D1	1:1000	Cell Signaling	sc-717
Cyclin E1	1:1000	Cell Signaling	sc-481
β-actin	1:2000	Sigma	A-5441

Hepatic cell lines and *in vitro* experiments

Cells were transfected for 72h with 50nM of LTβR siRNA (cat# SR302740) or scrambled negative control siRNA (SR30004) obtained from Origene (Rockville, MD), using Lipofectamine RNAiMAX reagent (Invitrogen) according to the manufacturer's protocol. LTβR stimulations were performed using agonistic anti-LTβR 4H8 at 2ug/mL for indicated durations.

Nanostring and PCR Analysis

Reverse Transcription-PCR was performed using the following human primers β-actin fwd 5'-AGAGCTACGAGCTGCCTGAC-3', β-actin rev 5'-AGCACTGTGTTGGCGTACAG-3' and LTβR primers from Bioneer (cat# P327477). GX mouse inflammatory kit was used with 20ng RNA/sample as per NanoString Technologies (Seattle, WA) instructions and normalized to internal controls with analyses performed using nsolver software (Seattle, WA). Total mRNA counts/20ng is indicated as “counts”.

Microarray analysis

A class comparison was performed to investigate differential gene expression between ICC vs. normal control. Changes in expression of genes at the $p<0.001$ level were considered to be statistically significant. Ingenuity Pathway Analysis (IPA) of the significant genes was performed to test for enriched signaling pathways. For Pearson correlation analysis of genes vs. LTBR gene expression, log 2 data of the mean for each gene from ICC samples was extracted and graphed in Graphpad Prism 5. Statistically significant changes in gene expression between ICC vs. Normal biliary epithelial cells (494 genes of 1,394 "proliferative class genes" from Llovet data set passed the $p<0.01$ test). Amongst the 494 genes are *Notch1, Hes1 and LTBR*. Hierarchal clustering (pearson correlation-complete linkage were performed with the three genes (Genesis v1.7.6,). (1)

1. Sturn A, Quackenbush J, Trajanoski Z. Genesis: cluster analysis of microarray data. Bioinformatics 2002;18:207-208.

Supplemental Figure 1. Expression of LTβR and its ligands in AKT/CAT-injected livers. A. mRNA expression (nanostring array) analysis of LTβR ligands was performed with RNA extracted from the livers day 40, post AKT/CAT injection. Total mRNA counts/20ng is indicated as "counts" B. Quantitative PCR (qPCR) analysis of LTβR mRNA levels from pT3 or AKT/CAT moribund livers. C. IHC to detect LTβR with AKT/CAT liver sections at day 14, 49 and 85. All scale bars depict mean values ±SEM. ns= not significant. Scale bars, 100 μm.

Supplemental Figure 2. LTβR-Fc treatment in AKT/CAT transfected mice failed to alter serum enzyme levels or lipogenic tumor morphology. Day 40 serum liver enzymes levels were measured from mice hydrodynamically transfected with AKT/CAT and treated with LTβR-Fc for 4 wks. B. Representative H&E staining from AKT/CAT moribund livers treated with LTβR-Fc.

Supplemental Figure 3. Sustained LTβR agonism alone is insufficient to drive hepatocyte proliferation. A. H&E and IHC staining for Ki-67, pAKT, and CAT with livers harvested at day 40 from mice hydrodynamically injected with pT3 empty plasmid and chronically administered anti-LTβR or Ig control for 4 weeks. B. Serum liver enzyme levels were measured at days 3, 10 and 30, post pT3 transfection following Ig or anti-LTβR treatment.

Supplemental Figure 4. Immunohistochemical staining characterizing AKT/CAT tumor-associated morphology. IHC staining was performed on liver sections from moribund AKT/CAT tumors using antibodies against α-feto protein (AFP), Glypican-3, CD34, Ki-67, cytokeratin (CK) 8, β-cat and pAKT. Lipid (Oil red O) and fibrosis (masson trichrome) staining was also performed.

Supplemental Figure 5. Evidence of cholangioblastic features in AKT/CAT hepatoblastomas. A. Representative H&E staining of AKT/CAT initiated tumors with cholangioblastic features and B. regions of desmoplasia (arrows).

Supplemental Figure 6. AKT/NICD induced ICC morphology. Representative H&E stained images form AKT/NICD tumor. Nodules form well defined ductular/pseudoglandular patterns with frequent appearance of mitotic figures (arrows).

Supplemental Figure 7. Expression of NF-κB p65 and c-MYC in AKT/CAT. A. Representative images of IHC staining using antibodies against NF-κB p65 at day 40 and moribund. B. c-MYC expression was measured from RNA isolated from AKT/CAT and AKT/NICD livers at day 40.

Supplemental Figure 8. Cholangiocyte proliferation/dysplasia selectively increased following AKT and LTβR activation. Cholangiocyte proliferation/dysplasia was defined by appearance of cholangiocyte proliferation, biliary dysplasia, bridging, however the lack of glandular patterns with histological scoring based on severity 1 (1-3) 2 (4-7) 3 (8-11) 4(>12). A. Representative H&E images from pT3, CAT, AKT, and AKT/CAT transfected mice administered anti-LTβR for 4 weeks. B. Histological scoring of H&E stained liver sections from day 40 and moribund AKT/CAT transfected livers.

Supplemental Figure 9. Immunohistochemical staining of human ICC. Representative IHC staining of ICC associated morphologies using antibodies against LTβR, LTβ, β-cat, pAKT, and Hes1 in five human ICC's. Arrows depict LTβ positive cells with leucocyte morphology.

Supplemental Figure 10. LTβ expression in AKT/NICD and cMET/CAT initiated hepatic tumors. A-B. mRNA expression (counts/20ng) of LTβR ligands was performed from liver RNA day 40 post pT3 empty vector, AKT/NICD or cMET/CAT transfection.

Supplemental Figure 11. LTβR signaling increases tumor CXCL10, CCL2 and immune infiltration. A. Day 40 quantitation of immune marker IHC (F4/80, CD3, B220) staining from pT3 or AKT/CAT-transfected mice following anti-LTβR or Ig

treatment was performed from at least 12 non-overlapping fields, n=3-4/group. B. Chemokine analysis of mRNA isolated from pT3, AKT/CAT or AKT/NICD transfected livers following treatment with anti-LTβR or Ig. n=3 or 4/group. All scale bars represent mean values ±SEM. Student T-test or Mann Whitney U was performed. *$p<0.05$, **$p<0.01$ *** and ****$p<0.001$.

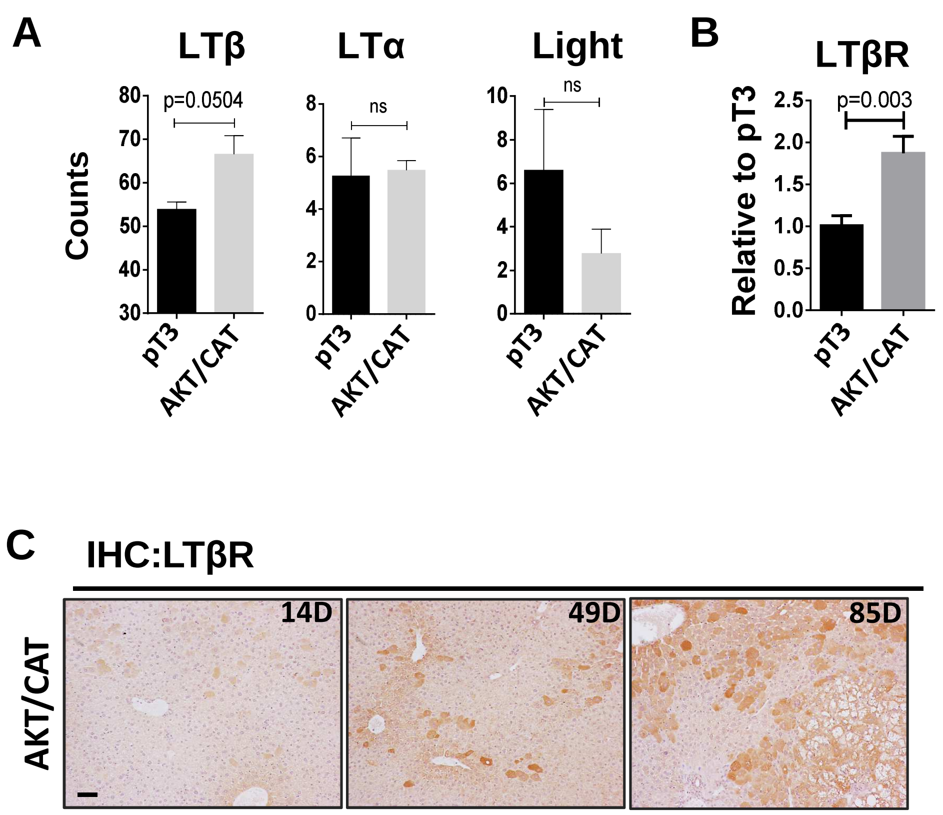

A
LTβ
LTα
Light
B
LTβR
Counts
p=0.0504
ns
ns
p=0.003
Relative to pT3
pT3
AKT/CAT
C
IHC:LTβR
AKT/CAT
14D
49D
85D

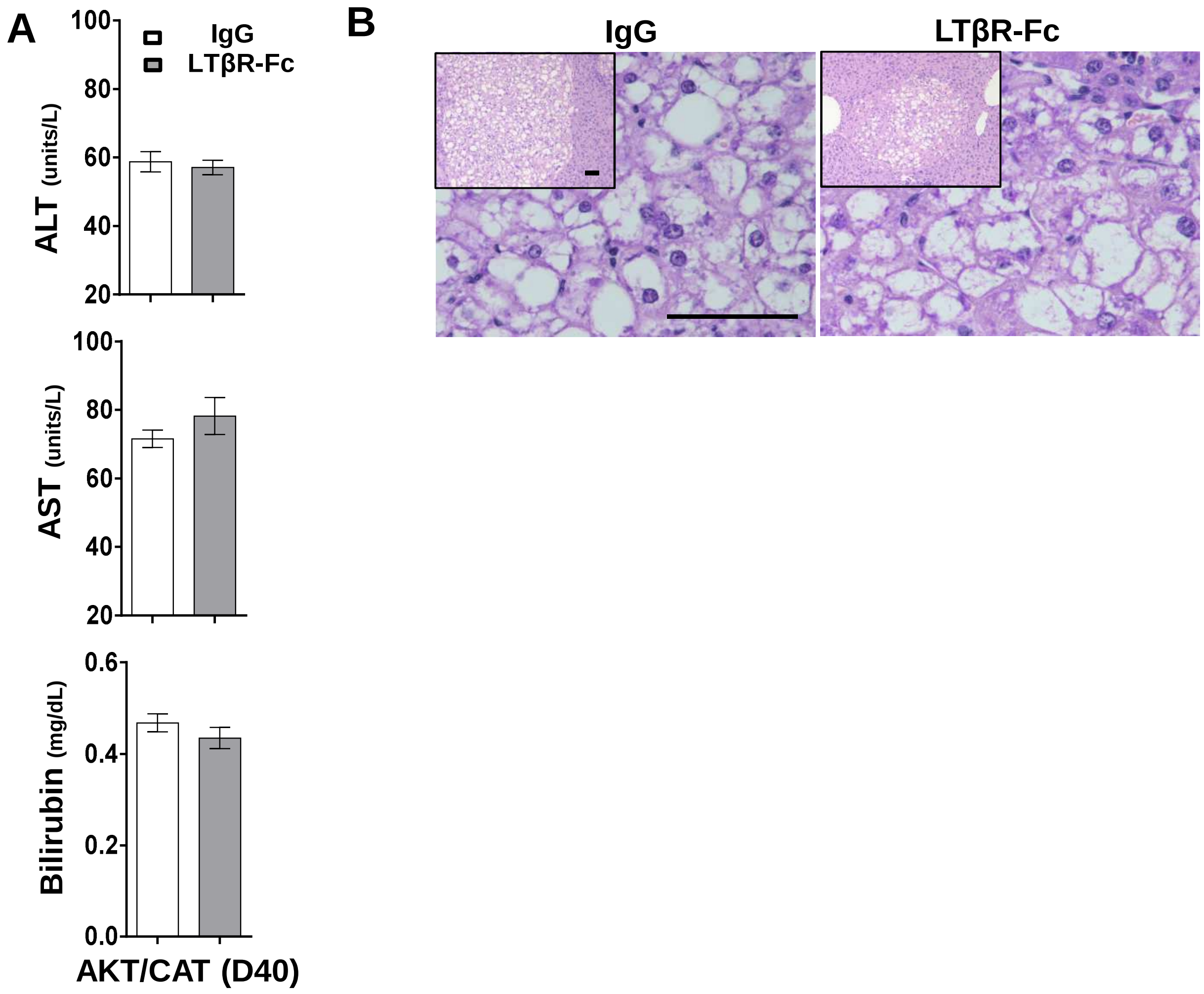

A
IgG
LTβR-Fc
ALT (units/L)
100
80
60
40
20
AST (units/L)
100
80
60
40
20
Bilirubin (mg/dL)
0.6
0.4
0.2
0.0
AKT/CAT (D40)
B
IgG
LTβR-Fc

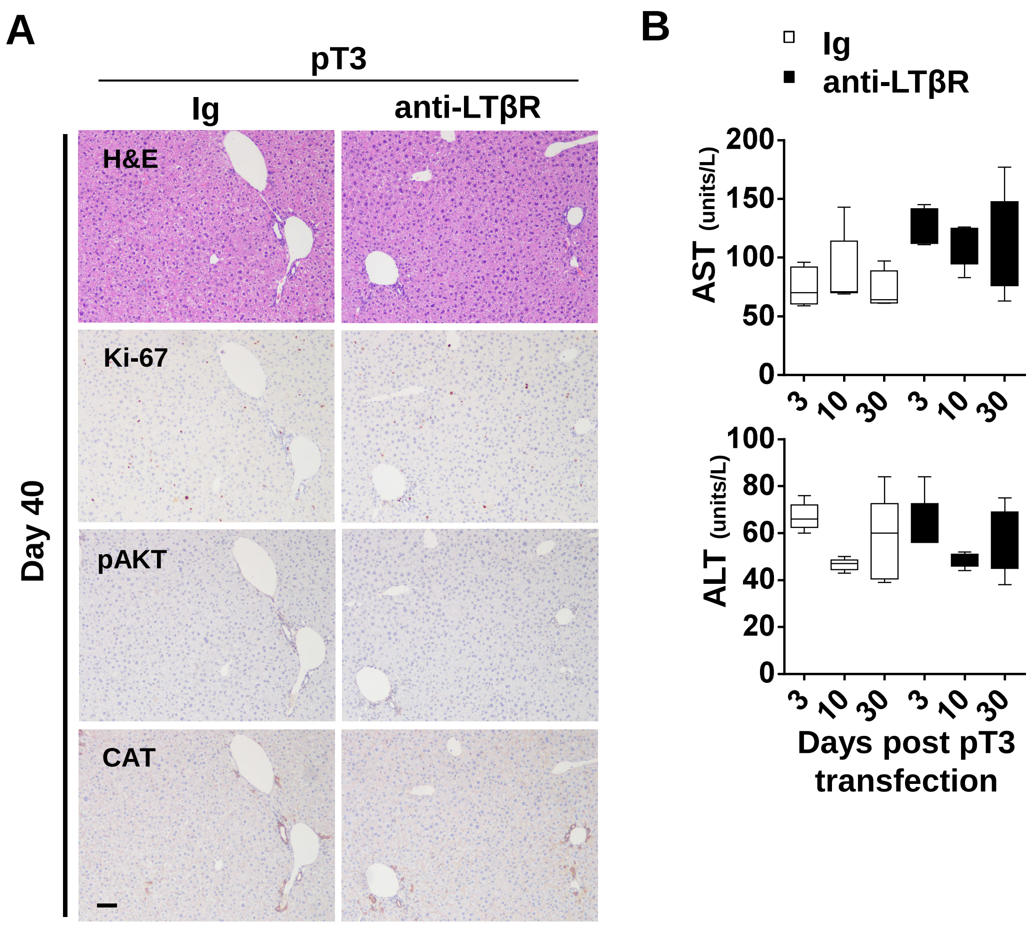
A
pT3
Ig
anti-LTβR
H&E
Ki-67
pAKT
CAT
Day 40
B
Ig
anti-LTβR
AST (units/L)
200
150
100
50
0
3
10
30
3
10
30
ALT (units/L)
100
80
60
40
20
0
3
10
30
3
10
30
Days post pT3 transfection

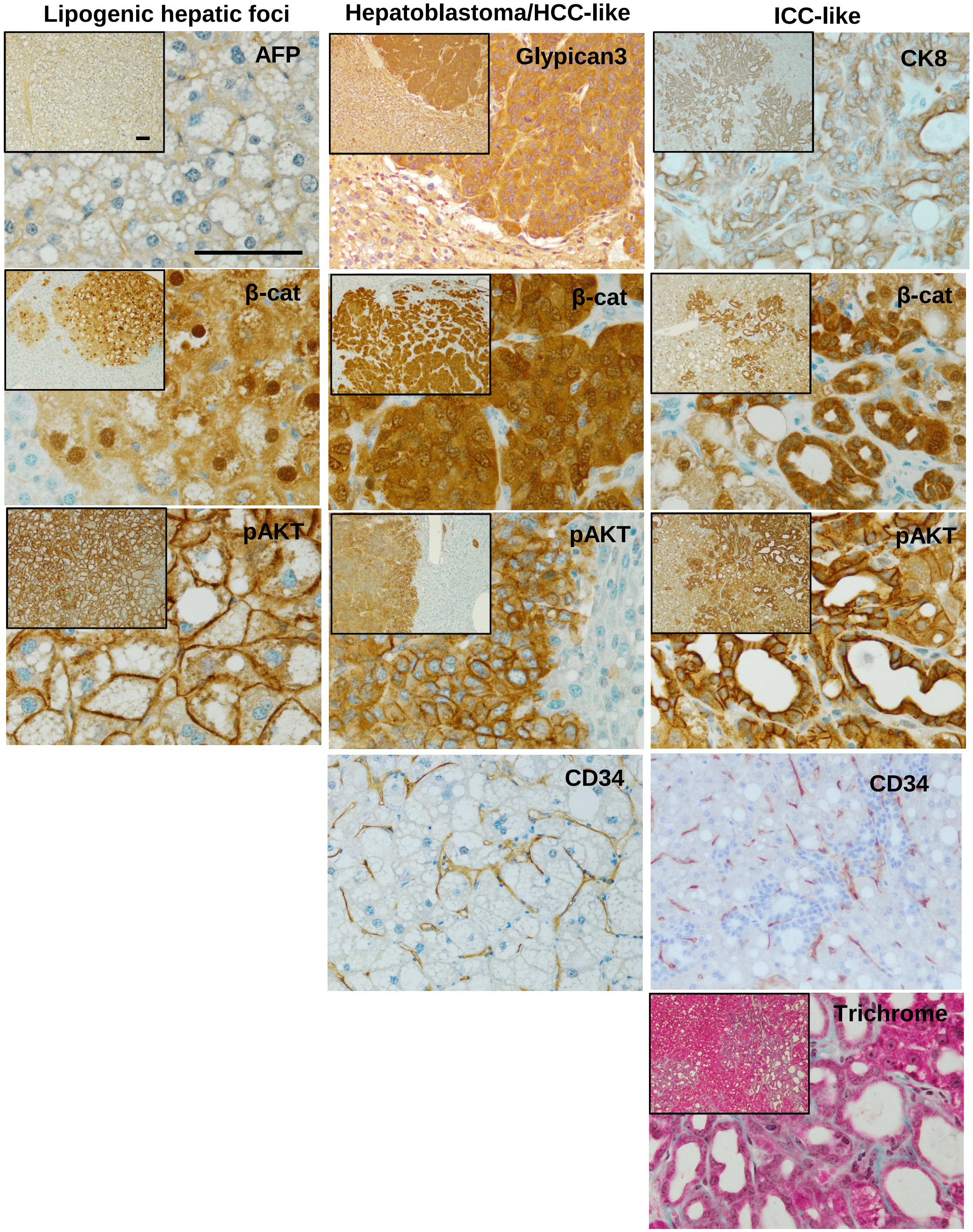

Lipogenic hepatic foci
Hepatoblastoma/HCC-like
ICC-like
AFP
Glypican3
CK8
β-cat
β-cat
β-cat
pAKT
pAKT
pAKT
CD34
CD34
Trichrome

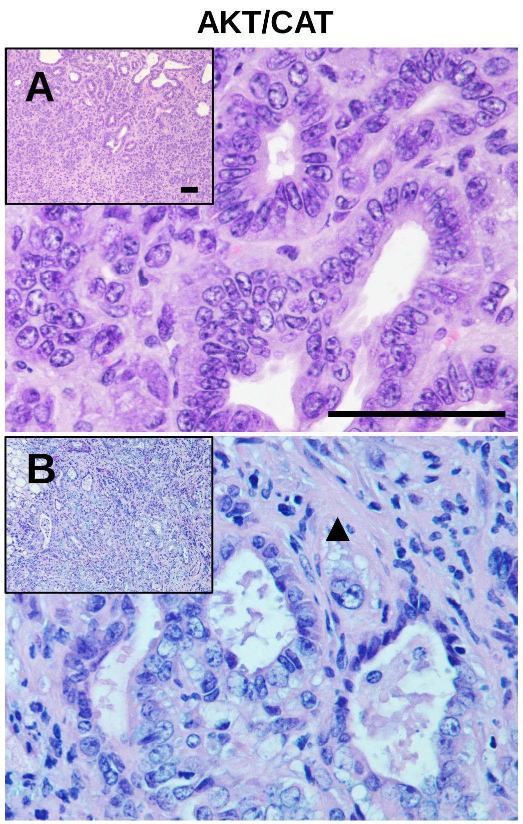
AKT/CAT
A
B

Mitotic spindles

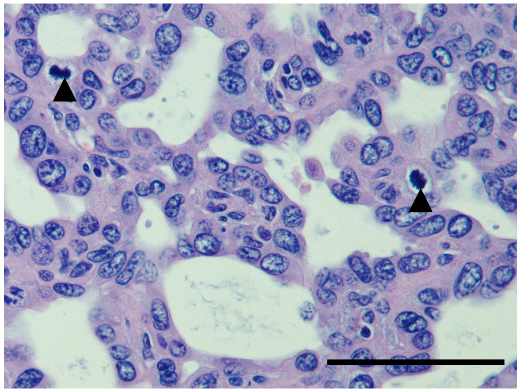

AKT/NICD

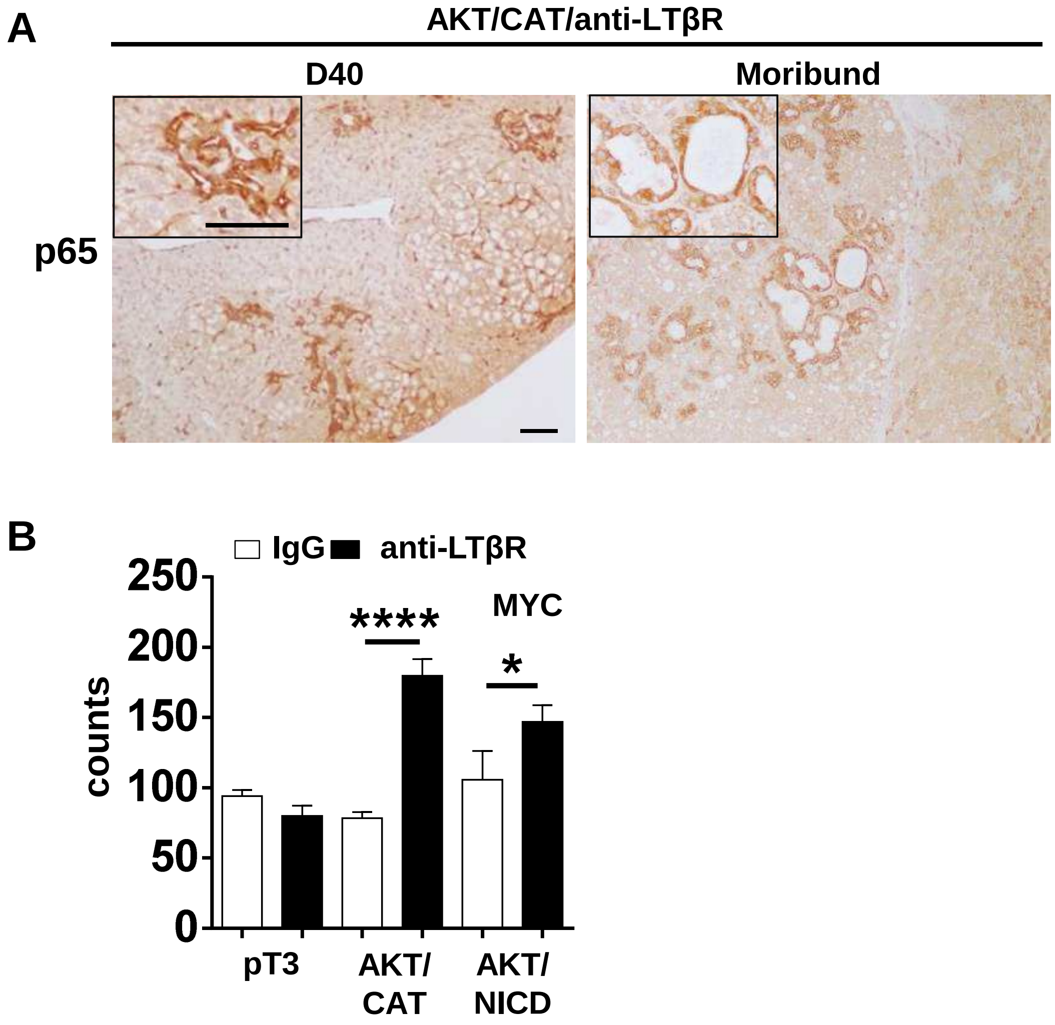
A
AKT/CAT/anti-LTβR
D40
Moribund
p65
B
IgG
anti-LTβR
MYC
****
*
250
200
150
100
50
0
counts
pT3
AKT/
CAT
AKT/
NICD

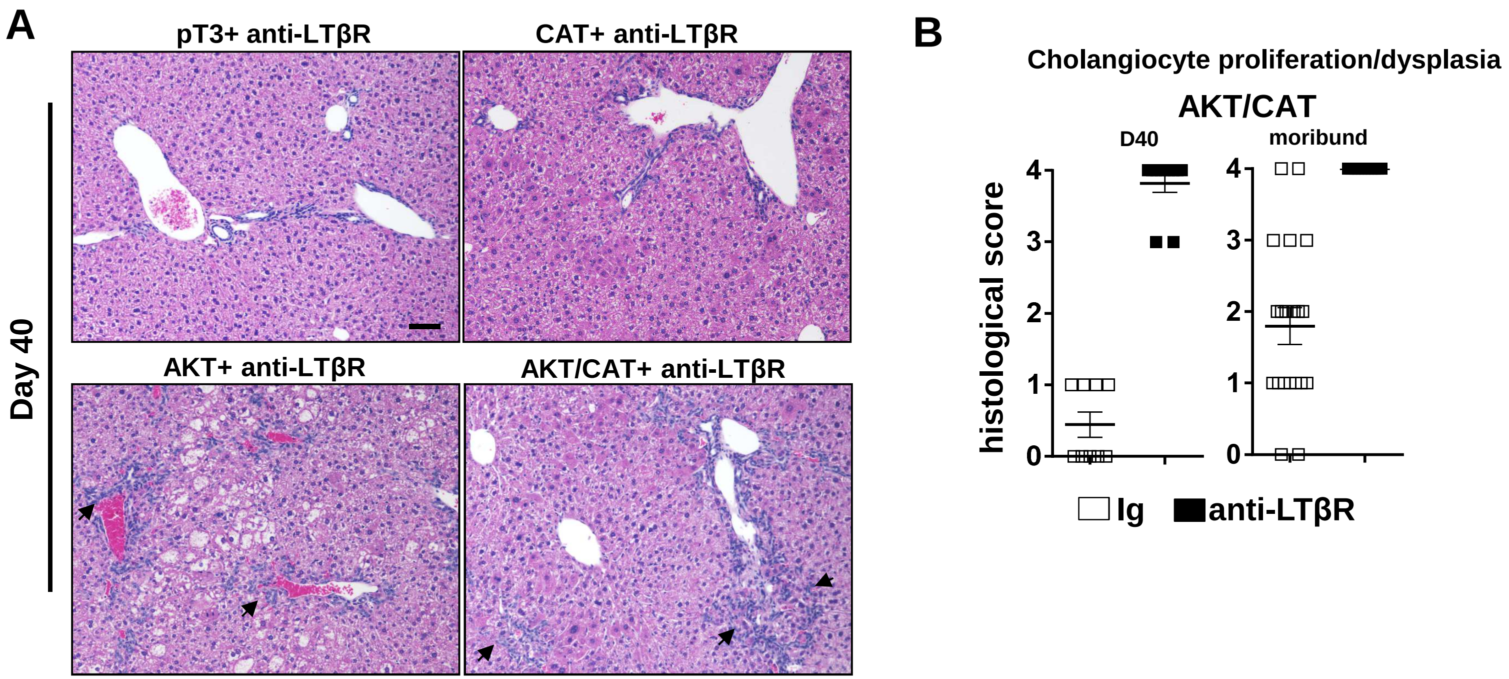
A
pT3+ anti-LTβR
CAT+ anti-LTβR
AKT+ anti-LTβR
AKT/CAT+ anti-LTβR
Day 40
B
Cholangiocyte proliferation/dysplasia
AKT/CAT
D40
moribund
histological score
0
1
2
3
4
Ig
anti-LTβR

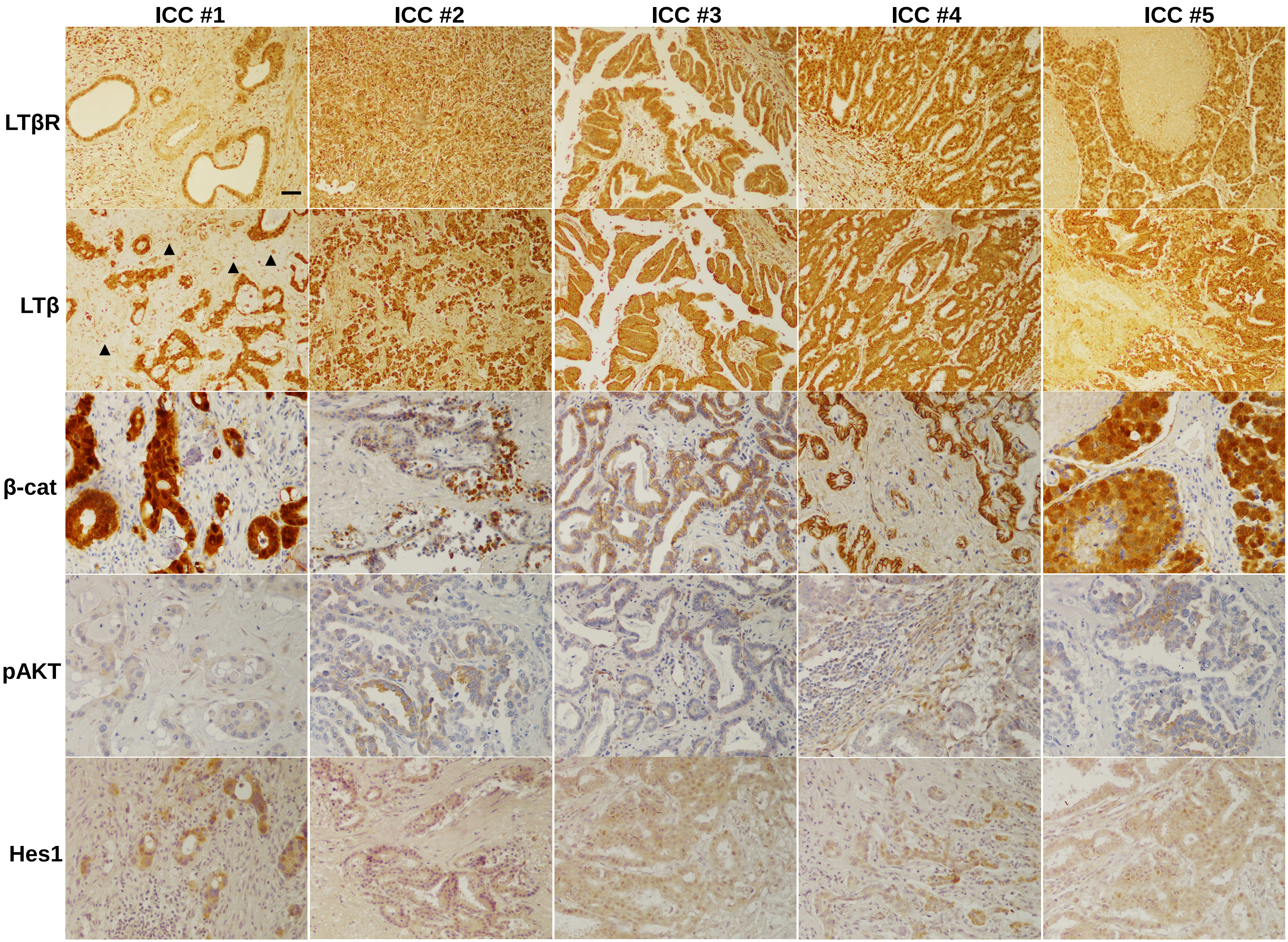

ICC #1
ICC #2
ICC #3
ICC #4
ICC #5
LTβR
LTβ
β-cat
pAKT
Hes1

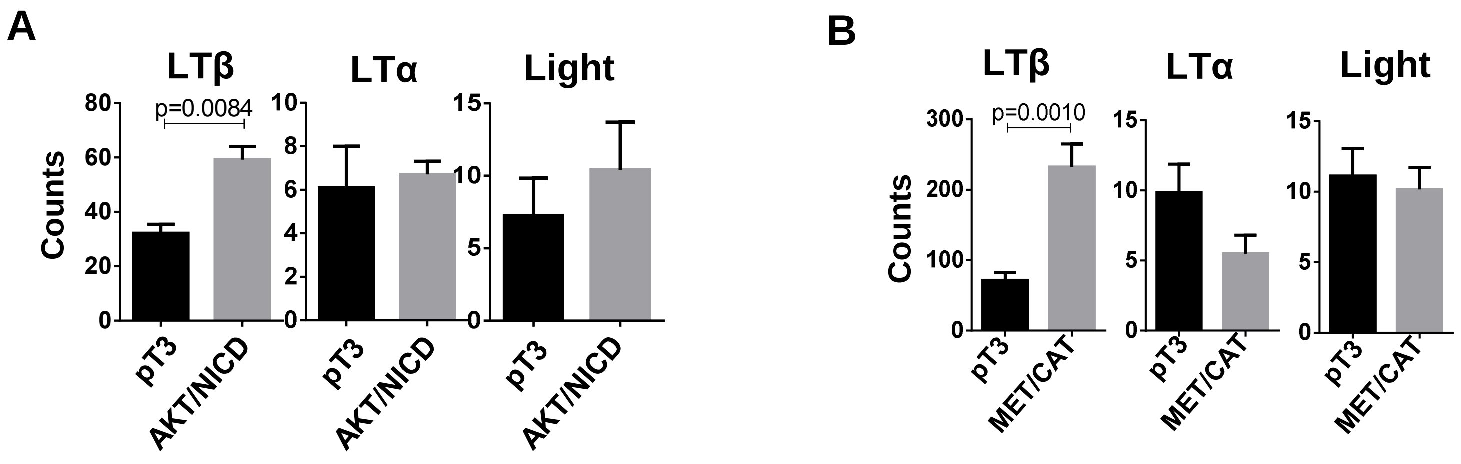
A
LTβ
LTα
Light
Counts
p=0.0084
pT3
AKT/NICD
B
LTβ
LTα
Light
Counts
p=0.0010
pT3
MET/CAT

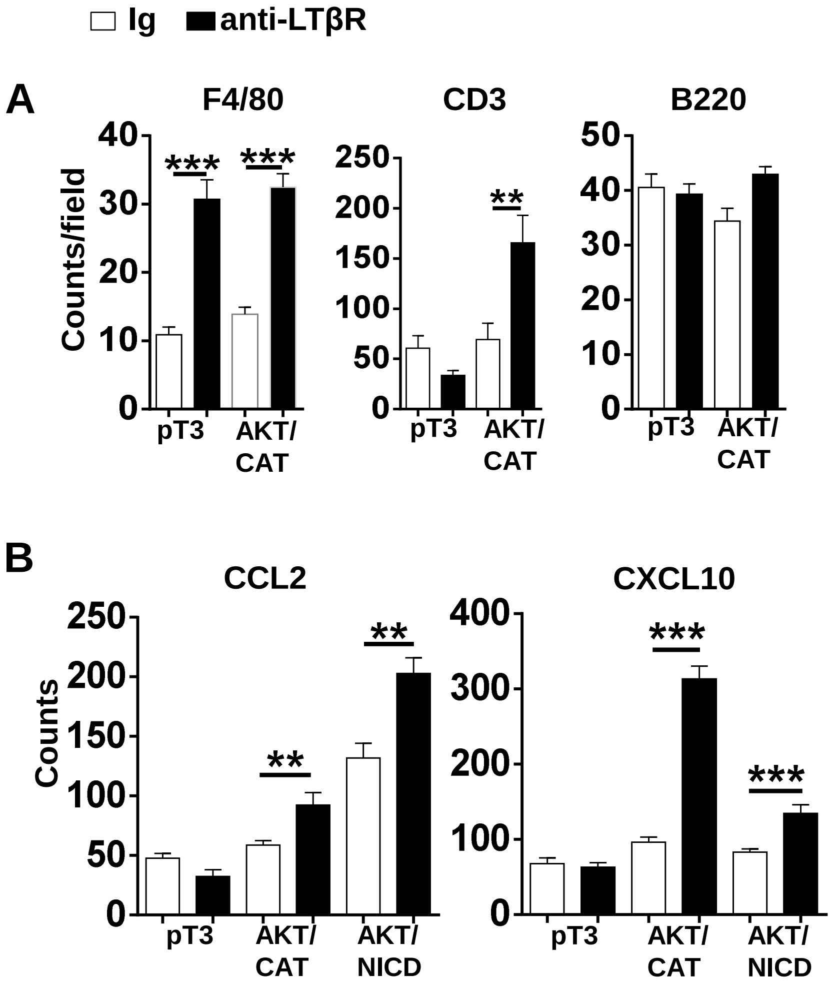

Ig
anti-LTβR
A
F4/80
CD3
B220
Counts/field
pT3
AKT/
CAT
B
CCL2
CXCL10
Counts
pT3
AKT/
CAT
AKT/
NICD
